# Hyperpalatable Foods Consumption in a Representative Sample of the General Population in Brazil: Differences of Binge and Non-Binge Eating Meals

**DOI:** 10.3390/bs13020149

**Published:** 2023-02-09

**Authors:** Carlos Eduardo Ferreira de Moraes, Phillipa Hay, Rosely Sichieri, Tera L. Fazzino, Carla Mourilhe, José Carlos Appolinario

**Affiliations:** 1 Group of Obesity and Eating Disorders (GOTA), Psychiatry Institute (IPUB), Federal University of Rio de Janeiro, Rio de Janeiro 21941-901, Brazil; 2 Translational Health Research Institute, School of Medicine, Western Sydney University, Sydney, NSW 2750, Australia; 3 Mental Health Services, South West Sydney Local Health District (SWSLHD), Campbelltown, NSW 2560, Australia; 4 Department of Epidemiology, Social Medicine Institute (IMS), State University of Rio de Janeiro (UERJ), Rio de Janeiro 28625-570, Brazil; 5 Department of Psychology, University of Kansas, Lawrence, KS 66045, USA; 6 Cofrin Logan Center for Addiction Research and Treatment, University of Kansas, Lawrence, KS 66045, USA

**Keywords:** binge eating disorder, bulimia nervosa, recurrent binge eating, food consumption, objective binge eating, subjective binge eating, 24 h food recall

## Abstract

The availability of hyper-palatable foods (HPF) increased over the past three decades worldwide, a period when eating disorders (ED) and obesity have become global public health concerns. The present study aimed to assess HPF consumption during binge and non-binge meals in a representative sample of adults with and without ED from a metropolitan city in Brazil. A total of 2297 individuals were interviewed in their homes by trained lay interviewers to assess the presence of binge eating disorder (BED), bulimia nervosa (BN), and recurrent binge eating (RBE). Information on their food consumption in objective and subjective binge eating episodes (OBE and SBE, respectively), as well as in the 24 h food recall were obtained. Individuals from the general population consumed 56% of their total calories from HPF. In non-binge meals, people with BN consumed substantially fewer calories from HPF than BED (63% vs. 48%) and RBE (63% vs. 48%) groups. During OBE, participants consumed an average of 70% of the calories from HPF, with no between-group differences. During SBE, subjects with BN consumed substantially fewer calories from HPF than those with BED (76% vs. 50%). In conclusion, HPF were highly consumed by the Brazilian population. However, there was a greater impact on BED and RBE subjects and during binge eating episodes.

## 1. Introduction

Eating disorders (ED) and obesity have become a worldwide public health concern in recent decades [1,2]. The point prevalence of ED raised from 3.5% between 2000 and 2006 to 7.8% in the 2013–2018 period [2]. Similarly, the proportion of adults with a body mass index (BMI) greater than 25 kg/m^2^ increased from 29% in 1980 to around 38% in 2013 [1]. Furthermore, individuals with ED comorbid with obesity are at a higher risk of clinical and psychosocial impairment [3]. Although these data came mostly from high-income countries, research in low/middle-income countries revealed a similar trend [4,5]. For example, in Brazil, two recent population-based studies found that the prevalence of binge eating spectrum conditions (binge eating disorder—BED; bulimia nervosa—BN; and recurrent binge eating—RBE) and obesity were 8.3% and 25.9%, respectively [6,7]. In addition, these conditions were associated with functional impairment, clinical and mental health issues, and socioeconomic inequalities [6,7,8].

Eating behavior can be triggered by metabolic reasons or hedonic drives [9]. For example, food intake is affected by appetite, energy demand, and other features such as taste, odor, and temperature of foods [10]. These processes are regulated by a complex interaction of hormones (e.g., leptin, ghrelin, and insulin) that are involved in the energy homeostasis in the central nervous system [11]. Similarly, these hormones play a role in the hedonic aspect of eating behaviors [12,13], that is, when people eat in response to cognitive, reward, and emotional factors (e.g., binge eating episodes) [9]. As these mechanisms can be affected by some characteristics of food (e.g., palatability and processing), assessing their impact on psychological and physiological processes has become an important research topic in obesity and ED areas [9,10,14,15].

Foods with higher amounts of sugar, sodium, and fat increase food intake and stimulate brain reward systems due to their enhanced palatability despite their caloric content [14,16]. In addition, these hyperpalatable foods (HPF) can be more attractive as they are quickly converted into energy [17]. Long-term consumption of HPF may impact signaling pathways related to food control, as they increase the expression of hunger and satiety signals but blunt or inhibit the signaling of some hormones that induce satiety (e.g., insulin and leptin) [10,16]. Further, there is an increased motivation to consume HPF. This process creates an artificially enhanced eating experience that promotes overeating [18]. For example, HPF are frequently consumed during binge eating episodes to distract individuals from their aversive emotions [19,20]. Furthermore, the consumption of HPF is associated with greater binge eating pathology [19] and obesity [10,16,21,22].

In recent decades, the availability of highly palatable foods increased worldwide [23,24]. Consequently, studying their impact on eating behavior became a public health issue. However, the existent literature focuses on specific nutrients (e.g., fat and sugar), characteristics (e.g., processing and energy density), and food groups (e.g., sweets and fried foods), limiting the comparability of the data [18,25,26,27]. To overcome this limitation, Fazzino and colleagues proposed a numeric classification for HPF, which is based on the amounts of fat, simple sugars, carbohydrates, and sodium [28]. It was validated in the US food system and has been used to assess HPF consumption in clinical and community settings [19,28]. Fazzino et al. found that the consumption of HPF (mainly those high in carbohydrates and sodium) was associated with greater caloric consumption and increased risk of weight gain in a sample of healthy adults after a 1-year follow-up [29]. Bjorlie et al. revealed that 95% of the calories of objective binge eating episodes came from HPF in people with BN [19]. Additionally, HPF consumption was associated with greater binge eating frequency [19]. Taken together, these findings highlight the impact of HPF on ED pathology and weight management.

ED are prevalent and impairing conditions associated with clinical and socioeconomic consequences. However, information on the food consumption of people with these conditions from general population samples in middle-income countries is limited. Additionally, there is a lack of studies comparing HPF consumption across ED categories and healthy subjects. Thus, we aimed to assess HPF consumption during binge and non-binge meals in a representative sample of adults with and without binge eating spectrum disorders from the general population of Rio de Janeiro, Brazil.

## 2. Materials and Methods

### 2.1. Participants and Procedures

This study is part of the Binge Eating in Rio Study, an in-person, population-based, household survey developed to assess the prevalence of BED and its correlates among a representative sample of the residents of Rio de Janeiro, the second largest city in Brazil. The survey was developed according to a stratified and clustered probability sample selected in three stages: census enumeration areas (CEAs), households and eligible adults (18–60 years old). Detailed information on the survey’s sampling procedures is reported by Appolinario et al. [6]. Pregnant and lactating women were excluded. The data collection was performed between September 2019 and February 2020.

### 2.2. Measures

#### 2.2.1. Diagnosis of Binge Eating Spectrum Disorders

In the first stage of the survey, 2297 participants completed the questionnaire on eating and weight patterns-5 (QEWP-5) for the screening of BED, BN, and RBE (Table 1). QEWP-5 is a self-report instrument developed to screen BED and BN according to DSM-5 criteria [30]. The Brazilian version of the questionnaire was validated in a sample from the general population and showed satisfactory psychometric properties [31]. For this study’s purposes, RBE was identified when individuals reported objective binge eating episodes at least once a week in the last three months, but they did not fulfill the full criteria for BED (i.e., at least three of five binge-eating-associated features and marked distress regarding the episode) [6].

In the second stage, a research assistant selected all screen-positive cases of BED, BN, and their subthreshold forms and a subset of the screen-negative cases. They were interviewed by telephone by two Ph.D. students (CEFM and CM) experienced in ED assessment to confirm BED and BN diagnoses. The interviews were conducted according to the eating disorders section of the structured clinical interview for DSM-IV (SCID-P) [32], adapted to DSM-5 [33]. SCID-P is the gold-standard method for the diagnosis of ED and was validated for administration by telephone [34]. All interviews were revised by a senior psychiatrist (JCA), experienced in the field of ED and who had not seen the QEWP-5 answers.

#### 2.2.2. Dietary Intake Assessment

Food consumption on regular meals and binge eating episodes was assessed through food recalls, which were performed on the day of the first stage of the survey, as described below.

##### Non-Binge Meals

We used a 24 h food recall to collect information about all foods and beverages consumed on the day before the screening interview. Information about the amount of food consumed, cooking methods (e.g., raw, cooked, or fried), and condiments or sugar/sweeteners added were also collected. These data were obtained through a software (ERICA-REC24h) validated for the assessment of food consumption in population-based epidemiological studies [35]. The interview technique used was the multiple-pass method, which consists of a five-step interview to reduce the underreporting of food intake. This software used the list of foods from the 2008–2009 Brazilian household budget survey [36].

##### Binge Eating Episodes

We collected data on food consumption during objective and subjective binge eating episodes. Objective binge eating episodes (OBE) are characterized by the consumption of excessive amounts of food in a discrete-time interval (<2 h) in which individuals experience a feeling of loss of control over eating [37]. Conversely, subjective binge eating episodes are defined by the feeling of loss of control during the consumption of small or moderate amounts of food [38]. Food consumption during binge eating episodes was assessed through a question from QEWP-5 about the characterization of a typical episode that occurred in the previous three months (e.g., “as best you can remember, please list everything you ate and drank during that episode. Please list the foods eaten and liquids consumed during the episode. Be specific—include brand names where possible and amounts or portion sizes as best you can estimate”). Interviewers followed the same steps employed in the 24 h food recall. However, for this purpose, ERICA-REC24h was adapted to assess the dietary intake during binge eating episodes. Previous research has used food recalls for the assessment of dietary intake and HPF consumption during binge eating episodes [19,39].

##### Hyperpalatable Food Classification

All foods reported in the 24 h and binge eating episode recalls were classified according to the HPF criteria proposed by Fazzino et al. [28]. Beverages were excluded from the analysis as the HPF definition does not apply to liquids [28]. The percentage of calories from fat, sugar, and carbohydrates (after subtracting sugar and fiber) of each food item was calculated. In addition, the percentage of sodium per food weight in grams was estimated. In the sequence, food items were classified as HPF when fulfilling one of the following criteria:

1. >25% of calories from fat and ≥0.30% sodium: for example, meats (e.g., bacon, hot dog) and meal-based items (e.g., pizza);
2. >20% of calories from fat and >20% of calories from sugar: for example, sweets and desserts (e.g., cake and ice cream);
3. >40% of calories from carbohydrates and ≥0.20% sodium: including bread, salty and savory snacks (e.g., crackers, popcorn), among others.

Evidence supports the convergent and divergent validity of such criteria in detecting foods that are expected to be hyperpalatable (e.g., fast foods and sweets) and non-identifying foods that are considered non-hyperpalatable [28].

#### 2.2.3. Sociodemographic and Clinical Variables

Sociodemographic characteristics included: age, sex, and ethnicity. They were obtained at the beginning of the household interview. Weight and height were measured at the household, using a digital scale with a maximum capacity of 150 kg and a precision of 100 g (Plenna^®^, São Paulo, Brazil). Height was measured using a portable stadiometer with a maximum range of 200 cm and a precision of 0.1 cm (model 206; Seca^®^, Hamburg, Germany). BMI was calculated and classified into the following categories: underweight (<18.5 kg/m^2^), normal weight (18.5 kg/m^2^–24.9 kg/m^2^), overweight (25 kg/m^2^–29.9 kg/m^2^), and obese (≥30 kg/m^2^) [40].

### 2.3. Data Analysis

Data are presented through weighted prevalence, means, standard error (SE), and 95% confidence intervals (95%CI). Information on the weighting approach has been reported elsewhere [6]. Demographics and metabolic characteristics according to ED status were tested by the Wald chi-square test based on the difference between observed and expected weighted frequencies. Regarding the analyses of the food consumption in the 24 h food recall and during binge eating episodes, the total caloric intake was calculated, and log transformed due to the skewed distribution of the values. The total calories from HPF (kcalHPF) and the contribution of HPF to the total energy intake (%HPF) were estimated. The kcalHPF was estimated by summing the amount of calories consumed from HPF. The %HPF was calculated as the kcalHPF divided by the caloric intake [28]. Furthermore, the proportion of calories from HPF derived from carbohydrates and lipids was estimated. The *t*-test was used to estimate differences within binge eating spectrum disorders (for OBE and SBE recalls) and between ED categories and those without ED (participants who did not engage in binge eating episodes) in the 24 h food recall. Statistically significant differences were reached at *p* < 0.05. All statistical analyses were performed considering weights and the complex design of the survey through Proc Survey procedures in the Statistical Analysis System (SAS), release 9.5 (SAS, 2003).

## 3. Results

### 3.1. Sample Characteristics

Of the 2297 participants of the study, 136 were diagnosed with binge eating spectrum disorders (29 with BED, 17 with BN, and 90 with RBE). Participants without ED (n = 2161) had a mean age of 38.3 (SE = 0.4) years and those with BED, BN, and RBE had a mean age of 40.3 (SE = 3.3), 31.9 (SE = 3.7) and 34.7 (SE = 1.4) years, respectively. There were no statistically significant differences in terms of age. Compared to the general population, BED, BN and RBE were significantly more prevalent in females 83.2%, 90.1% and 76.5% and in individuals with black ethnicity for BN 49.0%. Regarding BMI, there was a greater prevalence of obesity in people with binge eating spectrum disorders. Table 2 shows detailed information on the sociodemographic characteristics of our sample.

### 3.2. Energy Intake and HPF Consumption during Binge Eating Episodes

#### 3.2.1. Objective Binge Eating Episodes

Individuals with BED consumed a mean of 1184 kcal; those with BN consumed a mean of 1023 kcal, and participants with RBE consumed a mean of 994 kcal during the episodes. The percentage of energy from HPF during OBEs was 69% for those with BED, 62% for subjects with BN and 76% for individuals with RBE. The greatest part of the calories from HPF came from carbohydrates, regardless of the diagnosis. No differences were found between groups for any of the variables (Table 3).

#### 3.2.2. Subjective Binge Eating Episodes

Of the 136 participants diagnosed with binge eating spectrum conditions, 46 also engaged in SBE (nine with BED, 10 with BN, and 27 with RBE) (Table 4). Individuals with BED consumed a mean of 710 kcal; those with BN consumed a mean of 954 kcal, and participants with RBE consumed a mean of 639 kcal during the episodes. The percentage of energy from HPF during SBEs for BED and RBE subjects was substantially higher than for people with BN (BED vs. BN: 76% vs. 50%; RBE vs. BN: 72% vs. 50%). However, individuals with BN consumed a substantially higher percentage of the calories from HPF from carbohydrates than people with BED (72% vs. 46%). Conversely, subjects with BED consumed a substantially higher percentage of the calories from HPF from lipids than people with BN (36% vs. 20%). Nevertheless, the 95%CI did not show statistically significant differences due to the small sample size in the BN group (Table 4).

### 3.3. Energy Intake and HPF Consumption during Non-Binge Meals

In the 24 h food recall, the mean energy intake was 1793 kcal. People without ED consumed a mean of 1783 kcal, participants with BED consumed a mean of 2153 kcal, subjects with BN consumed a mean of 2219 kcal, and those with RBE ingested a mean of 1822 kcal. However, although participants with BED, BN, and RBE reported a higher energy intake compared to those without ED, there were no statistically significant differences between groups. The proportion of energy consumed from HPF during non-binge meals was 56% for those without ED, 63% for people with BED, 44% for subjects with BN, and 63% for individuals with RBE. The percentage of energy consumed from HPF by participants with BED and RBE was substantially higher than those with BN and people without ED. However, the 95%CI did not show statistically significant differences due to the large difference in the sample sizes between the groups (Table 5).

## 4. Discussion

To our knowledge, this is the first investigation of the hyper-palatable nature of eating in both the general population and in people with binge eating spectrum conditions from a representative community sample of a metropolitan city of a middle-income country. We found that people from the general population consumed a high percentage of calories from HPF. Similarly, subjects with BED and RBE consumed a greater proportion of HPF during non-binge meals. Conversely, individuals with BN restricted their HPF consumption compared to other groups. During OBEs, people with binge eating spectrum conditions consumed a high percentage of calories from HPF, regardless of the diagnosis. Finally, during SBEs, subjects with BN consumed fewer calories from HPF than those with BED and RBE. Taken together, these findings highlight that HPF substantially impact the dietary intake of the Brazilian population, mostly in individuals with BED and RBE, as well as during binge eating episodes.

Our results indicate a high consumption of HPF by people without ED in the general population of Rio de Janeiro, Brazil. Although the lack of studies assessing the consumption of HPF in middle-income countries through a standardized definition limits the comparison of our findings, previous research has revealed an increase in the consumption of high-sugar, high-fat, and salty foods in Brazil [41,42]. For example, Sichieri et al., in 2015, found that sweets and cookies were the main contributors to the total energy intake of a representative sample of adolescents and adults [41]. Rodrigues et al. compared the quality of the Brazilian diet between 2008 and 2018 and found that there was an increase in the consumption of sandwiches [42]. Furthermore, cookies, savory snacks, and sweets were among the most consumed food items [42]. These findings highlight the frequent consumption of highly palatable and ready-to-eat foods by the Brazilian general population, which may contribute to the increasing rates of higher BMI, weight-related problems, and diet-related non-communicable diseases observed in such context [7,43].

In addition, our data are consistent with Bjorlie et al. [19], who found that people with BN restrict hyper-palatable foods in non-binge meals. They assessed a community sample of individuals with full-threshold and subthreshold BN and compared their food consumption during binge eating and restrictive episodes. Participants consumed a median of 2134 kcal during their objective binge eating episodes. Of those, 1846 kcal (95%) came from hyper-palatable foods. Conversely, the total energy intake in the restrictive episode was 558 kcal, with a median of 280 kcal from hyper-palatable foods, which comprised 61% of the total caloric consumption. Thus, the median and the percentage of calories from hyper-palatable foods were significantly lower during the restrictive episodes compared to objective binge eating episodes [19].

This study fulfilled a gap in the literature on the nutritional characteristics of SBE in the general population of middle-income countries. In our sample, people with binge eating spectrum conditions consumed around 700 kcal in their SBE. Furthermore, such episodes were mostly comprised of hyper-palatable foods, except for subjects with BN, which restricted their consumption compared to BED and RBE groups. Our data on SBE size are consistent with the findings of Bartholome et al. [44], which reported an average consumption of 680 kcal in a clinical sample of women with BED and obesity in the United States. Moreover, around 85% of these calories came from carbohydrates and fats. Additionally, sweets and salty snacks were the most consumed food in the SBE [44]. Taken together, these findings may suggest that HPF were primarily targeted for consumption during SBE in BED subjects. However, its lack of use of a standardized numeric definition for hyper-palatable foods limits its comparability to our results.

We found that a great part of the calories of the objective binge eating episodes came from hyper-palatable foods, irrespective of the diagnosis. Although this is consistent with previous research, the average binge eating size in our sample (1067 kcal) was lower than the values reported in clinical and laboratory studies from high-income countries (around 1800 kcal and 2500 kcal, respectively) [19,45]. However, these differences should be analyzed considering some circumstances. The DSM-5 does not establish a minimum threshold of calories for “a definitely large amount of food”. This aspect may vary according to sex, weight status, cultural and socioeconomic particularities across the study’s backgrounds [46,47,48,49,50,51]. For example, Forney et al. aimed to define the empirical threshold for a large amount of foods that are usually consumed during binge eating episodes. The authors revealed that the upper limits for a “normal consumption” of HPF, such as chocolates, sweets, and hamburgers, ranged from 400 kcal to 650 kcal for women. In contrast, these thresholds were between 466 and 800 kcal for men [51]. Therefore, the mean objective binge eating size reported in our study is in line with what has been considered a “large amount of food” in the ED literature. Finally, it is noteworthy to clarify that data on food consumption during binge eating episodes came mostly from clinical samples from the USA. Nevertheless, previous research revealed differences in food preferences between the Brazilian and American populations [50]. While Americans consume more frequently energy-dense foods, such as donuts, savory snacks, fast food, and desserts, Brazilians consume more dinner foods, such as rice, beans, and meats, which are less energy-dense [52].

Our results have some limitations. First, our sample is composed of individuals from the general population of a metropolitan city in Brazil. Thus, the generalization of the results to different settings (e.g., other Brazilian cities, rural areas, and other countries) is limited. Second, participants could have underreported their food consumption during both binge and non-binge meals due to embarrassment regarding the amount of food eaten during binge eating episodes or recall bias [53]. This is a common limitation in studies assessing food consumption through dietary recalls [54]. Some characteristics have been associated with greater underreporting, including sex (female), irregular eating habits, smoking, higher BMI, and low education [55,56]. However, the food recall is considered the most feasible self-report measure of energy intake [56] and has been frequently used in population-based studies (e.g., national dietary surveys) [57,58], as well as for the assessment of caloric intake during binge eating episodes [19,39]. Furthermore, it was validated for use in the Brazilian population and other countries [55,59]. Third, we assessed only one day of food recall due to operational issues. Therefore, our findings are not representative of the usual food consumption of the participants of the study. However, the mean energy intake of our sample was around 1800 kcal, which is similar to what has been reported in the most recent Brazilian national dietary survey (i.e., 1748 kcal) [60]. Fourth, the large differences in the group sizes associated with the great variability in energy intake may have underpowered the comparisons as the 95%CI do not show statistically significant differences. However, percentages of energy from HPF are more reliable because variations in both HPF and total energy intake cancel out and indicate the qualitative variation between diagnosed groups. The present study has, however, the following strengths: (1) The use of trained lay interviewers in the data collection; (2) The two-stage design to assess ED diagnosis, including the screening through the QEWP-5 and the use of SCID-P administered by clinicians specialized in ED assessment and treatment, under the revision of a senior expert in this field; (3) The use of a quantitative and standardized definition of HPF [28]; and (4) The inclusion of a non-ED group to compare the food consumption during non-binge meals.

These findings may have some clinical implications. Around 60% of the calories consumed by individuals from the general population in their regular meals were derived from HPF and were high in carbohydrates. Similarly, subjects with BED and RBE reported a high consumption of carbohydrates from HPF in both binge and non-binge meals. This eating pattern reflects a greater consumption of snack items, which are HPF high in carbohydrates and sodium [28] and may contribute to weight gain [61]. Thus, these individuals may need a more balanced diet, increasing the consumption of non-hyper-palatable and high-fiber foods such as fruits, vegetables, and whole wheat cereals. Conversely, people with BN would benefit from nutritional counseling to encourage the moderate consumption of HPF in their daily routine, as eating restrictions may contribute to binge eating episodes.

Future research is needed to investigate HPF consumption during binge and non-binge meals in broader populations, other ethnicities, in representative samples from other low- and middle-income countries, and in individuals with other common eating disorders (e.g., purging disorder). Additionally, the assessment of HPF consumption through two or more days of food recall and with larger ED samples would reduce the variability of the results and increase the power to compare the differences in the usual dietary habits across diagnoses. Furthermore, examining the availability of HPF in the Brazilian food system would be important to show the degree to which the Brazilian population is exposed to foods that are associated with overeating and higher weight.

## 5. Conclusions

People from the Brazilian general population showed high consumption of HPF in the 24 h food recall. Regarding subjects with binge eating spectrum conditions, those with BN consumed substantially fewer calories from HPF than participants with BED and RBE in non-binge meals. Regarding binge eating episodes, there was high consumption of HPF during OBEs, irrespective of diagnosis. However, during SBEs, participants with BN consumed substantially fewer calories from HPF than BED and RBE groups.

## Figures and Tables

**Table 1:** Diagnostic criteria for binge eating disorder (BED), bulimia nervosa (BN), and recurrent binge eating (RBE).

Criteria	BED	BN	RBE
OBE	Yes	Yes	Yes
Compensatory behaviors *	No	Yes	-
Frequency of OBE or Compensatory behaviors	≥1 x/wk	≥1 x/wk	≥1 x/wk
≥3 of 5 binge-eating-associated features +	Yes	-	No
Marked distress Regarding binge eating	Yes	-	No
Overvaluation of weight and shape	-	Yes	-

Note: * Self-induced vomit, excessive exercise, fasting, misuse of diuretics, laxatives, or other medications. + Eating faster than usual, eating until feeling uncomfortably full, eating large amounts of food when not feeling hungry, eating alone because of a feeling of embarrassment by the amount of food eaten, feeling disgusted with yourself/depressed/very guilty.

**Table 2:** Prevalence of binge eating spectrum disorders according to sociodemographic characteristics and weight status (*n* = 2297).

Variables	No ED			BED			BN			RBE		
	*n*	%	95%CI	*n*	%	95%CI	*n*	%	95%CI	*n*	%	95%CI
Total	2161	93.8	92.2–95.4	29	1.4	0.8–2.4	17	0.7	0.3–1.5	90	6.2	3.1–5.3
Sex												
Male	864	46.8	44.1–49.5	**5**	**0.5**	**0.2–1.3**	**2**	**0.1**	**0.03–0.6**	**19**	**2.4**	**1.4–4.0**
Female	1297	47.0	44.3–49.8	**24**	**2.3**	**1.2–4.2**	**15**	**1.3**	**0.5–2.9**	**71**	**5.6**	**4.1–7.6**
Race/skin color												
White	862	35.5	32.1–38.9	6	0.8	2.9–2.1	6	0.5	0.2–1.2	34	3.6	2.3–5.4
Black	371	17.8	15.4–20.2	7	2.4	0.9–6.3	**6**	**1.8**	**0.5–5.9**	18	4.2	2.1–7.9
Mixed ^a^	928	40.5	37.7–43.3	16	1.5	0.8–2.8	5	0.5	0.2–1.2	38	4.4	3.1–6.3
Age												
18 to 30 years	524	29.9	25.8–33.9	6	1.1	0.4–2.9	5	1.0	0.2–3.9	32	5.3	3.4–8.2
31 to 45 years	746	34.2	31.1–37.4	12	1.3	0.7–2.4	10	1.0	0.4–2.4	33	3.8	2.5–5.6
46 to 60 years	891	29.7	27.1–32.3	11	1.8	0.7–4.4	2	0.1	0.03–0.7	25	3.1	1.7–5.1
BMI ^b^												
Underweight	59	3.4	2.3–4.5	0	–		0	–	–	3	7.0	1.7–24.8
Normal Weight	666	30.8	27.6–33.9	**1**	**8.1**	**1.1–41.6**	**2**	**7.7**	**1.6–30.1**	**16**	**18.2**	**10.5–29.5**
Overweight	790	35.1	31.9–38.4	**6**	**27.1**	**9.1–57.9**	**4**	**18.2**	**4.0–54.0**	**24**	**21.8**	**13.5–33.1**
Obese	531	24.5	21.9–27.1	**21**	**64.8**	**36.1–85.7**	**11**	**74.1**	**40.5–92.3**	**46**	**53.0**	**40.2–65.5**

Note: No ED = no eating disorders; BED = binge eating disorder (DSM-5); BN = bulimia nervosa (DSM-5), RBE = recurrent binge eating (≥1 binge eating episode/wk. in the last 3 mo.). ^a^ Mixed: brown, yellow, and indigenous; ^b^ BMI = weight(kg)/height(m^2^). Results in bold are statistically significant at a *p* < 0.05.

**Table 3:** Total energy intake, energy from hyper-palatable foods and consumption of carbohydrates and lipids from HPF during objective binge eating episodes across binge eating spectrum disorders.

OBE	BED (*n* = 29)		BN (*n* = 17)		RBE (*n* = 90)		BED vs. BN BED vs. RBE BN vs. RBE	
	Mean (SE)	95%CI	Mean (SE)	95%CI	Mean (SE)	95%CI	* *t*	*p*
Total kcal/OBE	1184 (145.5)	891–1476	1023 (170.5)	680–1365	994 (100.1)	793–1195	0.6 1.1 0.1	0.53 0.27 0.89
HPF kcal/OBE	882 (150.1)	581–1184	688 (175.6)	336–1041	790 (91.4)	606–973	0.7 0.5 −0.5	0.45 0.60 0.63
% kcal from HPF/OBE	69	57–82	62	49–75	76	69–83	0.5 −0.9 −1.7	0.59 0.38 0.10
% of kcal from HPF from carbohydrates/ OBE	58	46–69	54	43–65	54	50–58	0.7 0.9 −0.2	0.49 0.37 0.85
% of kcal from HPF from lipids/ OBE	29	20–38	34	21–46	32	28–35	−0.2 −0.5 −0.02	0.81 0.64 0.98

Notes: * *t*-test; OBE: objective binge eating episodes; BED: binge eating disorder; BN: bulimia nervosa; RBE: recurrent binge eating; SE: standard error; CI: confidence interval; HPF: hyper-palatable foods.

**Table 4:** Total energy intake, energy from hyper-palatable foods and consumption of carbohydrates and lipids from HPF during subjective binge eating episodes across binge eating spectrum disorders.

SBE	BED (*n* = 9)		BN (*n* = 10)		RBE (*n* = 27)		BED vs. BN BED vs. RBE BN vs. RBE	
	Mean (SE)	95%CI	Mean (SE)	95%CI	Mean (SE)	95%CI	* *t*	*p*
Total kcal/SBE	710 (127.3)	399–1022	954 (217.5)	422–1487	639 (57.9)	497–780	−1.1 0.3 1.4	0.30 0.78 0.20
HPF kcal/SBE	466 (34.4)	382–550	461 (89.9)	241–681	439 (54.9)	305–574	0.4 0.7 0.1	0.71 0.51 0.93
% of kcal from HPF/SBE	76	54–98	50	29–72	72	55–90	2.0 0.4 −1.7	0.08 0.72 0.15
% of kcal from HPF from carbohydrates/ SBE	46	36–56	72	56–88	54	42–67	−3.1 −1.3 1.5	0.03 0.23 0.20
% of kcal from HPF from lipids/SBE	36	30–43	20	7–32	29	21–37	2.9 2.1 −1.2	0.03 0.08 0.27

Notes: * *t*-test; SBE: subjective binge eating episodes; BED: binge eating disorder; BN: bulimia nervosa; RBE: recurrent binge eating; SE: standard error; CI: confidence interval; HPF: hyper-palatable foods.

**Table 5:** Total energy intake, energy from hyper-palatable foods and consumption of carbohydrates and lipids from HPF in the 24 h food recall.

Variable	No ED (*n* = 2048)		BED (*n* = 29)		BN (*n* = 16)		RBE (*n* = 81)		No ED vs. BED No ED vs. BN No ED vs. RBE		BED vs. BN BED vs. RBE BN vs. RBE	
	Mean (SE)	95%CI	Mean (SE)	95%CI	Mean (SE)	95%CI	Mean (SE)	95%CI	t *	*p*	t *	*p*
Total kcal	1783 (47.1)	1690–1876	2153 (365.2)	1430–2876	2219 (700.1)	832–3607	1822 (163.1)	1499–2145	0.7 −0.7 −0.2	0.49 0.50 0.85	0.8 0.7 −0.6	0.43 0.46 0.54
HPF kcal	1102 (42.6)	1018–1187	1480 (319.2)	848–2112	1492 (578.8)	346–2638	1229 (144.6)	493–1516	1.4 −1.1 1.2	0.15 0.27 0.23	1.4 0.7 −1.3	0.16 0.48 0.18
% of kcal from HPF	56	55–57	63	57–69	48	36–59	63	58–68	2.9 −1.4 2.9	0.01 0.17 0.01	2.1 0.3 −2.1	0.03 0.79 0.04
% of kcal from HPF from carbohydrates	56	55–57	57	52–62	48	38–58	58	53–61	0.6 −1.3 1.2	0.55 0.19 0.23	1.4 −0.3 0.6	0.16 0.75 0.14
% of kcal from HPF from lipids	28	27–29	30	26–33	35	27–43	27	25–29	1.0 1.9 −0.2	0.35 0.06 0.87	−1.0 1.0 1.8	0.31 0.33 0.07

Notes: * *t*-test; missing values not included (*n* = 123; 5.4%); No ED = no eating disorders; BED: binge eating disorder; BN: bulimia nervosa; RBE: recurrent binge eating; SE: standard error; CI: confidence interval; HPF: hyper-palatable foods.

## Data Availability

The data presented in this study are available on request from the corresponding author.

## References

[1] Ng M., Fleming T., Robinson M., Thomson B., Graetz N., Margono C., Mullany E.C., Biryukov S., Abbafati C., Abera S.F. (2014). Global, Regional, and National Prevalence of Overweight and Obesity in Children and Adults during 1980–2013: A Systematic Analysis for the Global Burden of Disease Study 2013. Lancet.

[2] Galmiche M., Déchelotte P., Lambert G., Tavolacci M.P. (2019). Prevalence of Eating Disorders over the 2000–2018 Period: A Systematic Literature Review. Am. J. Clin. Nutr..

[3] da Luz F.Q., Hay P., Touyz S., Sainsbury A. (2018). Obesity with Comorbid Eating Disorders: Associated Health Risks and Treatment Approaches. Nutrients.

[4] Kolar D.R., Rodriguez D.L.M., Chams M.M., Hoek H.W. (2016). Epidemiology of Eating Disorders in Latin America: A Systematic Review and Meta-Analysis. Curr. Opin. Psychiatry.

[5] Kessler R.C., Berglund P.A., Chiu W.T., Deitz A.C., Hudson J.I., Shahly V., Aguilar-gaxiola S., Alonso J., Angermeyer M.C., Benjet C. (2013). The Prevalence and Correlates of Binge Eating. Biol. Psychiatry.

[6] Appolinario J.C., Sichieri R., Lopes C.S., Moraes C.E., da Veiga G.V., Freitas S., Nunes M.A.A., Wang Y.P., Hay P. (2022). Correlates and Impact of DSM-5 Binge Eating Disorder, Bulimia Nervosa and Recurrent Binge Eating: A Representative Population Survey in a Middle-Income Country. Soc. Psychiatry Psychiatr. Epidemiol..

[7] de Souza Ferreira A.P., Szwarcwald C.L., Damacena G.N., de Souza Júnior P.R.B. (2021). Increasing Trends in Obesity Prevalence from 2013 to 2019 and Associated Factors in Brazil. Rev. Bras. Epidemiol..

[8] Triaca L.M., dos Santos A.M.A., Tejada C.A.O. (2020). Socioeconomic Inequalities in Obesity in Brazil. Econ. Hum. Biol..

[9] Berthoud H.R. (2011). Metabolic and Hedonic Drives in the Neural Control of Appetite: Who Is the Boss?. Curr. Opin. Neurobiol..

[10] de Macedo I.C., de Freitas J.S., da Silva Torres I.L. (2016). The Influence of Palatable Diets in Reward System Activation: A Mini Review. Adv. Pharmacol. Sci..

[11] Small C.J., Bloom S.R. (2004). Gut Hormones and the Control of Appetite. Trends Endocrinol. Metab..

[12] Dardeno T.A., Chou S.H., Moon H.S., Chamberland J.P., Fiorenza C.G., Mantzoros C.S. (2010). Leptin in Human Physiology and Therapeutics. Front. Neuroendocrinol..

[13] Atalayer D., Gibson C., Konopacka A., Geliebter A. (2013). Ghrelin and Eating Disorders. Prog. Neuropsychopharmacol. Biol. Psychiatry.

[14] Wang G.J., Volkow N.D., Telang F., Jayne M., Ma J., Rao M., Zhu W., Wong C.T., Pappas N.R., Geliebter A. (2004). Exposure to Appetitive Food Stimuli Markedly Activates the Human Brain. Neuroimage.

[15] Leigh A.S., Morris M.J. (2018). The Role of Reward Circuitry and Food Addiction in the Obesity Epidemic: An Update. Biol. Psychol..

[16] Erlanson-Albertsson C. (2005). How Palatable Food Disrupts Appetite Regulation. Basic Clin. Pharmacol. Toxicol..

[17] Nesse R.M., Berridge K.C. (1997). Psychoactive Drug Use in Evolutionary Perspective. Science.

[18] Morris M.J., Beilharz J.E., Maniam J., Reichelt A.C., Westbrook R.F. (2015). Why Is Obesity Such a Problem in the 21st Century? The Intersection of Palatable Food, Cues and Reward Pathways, Stress, and Cognition. Neurosci. Biobehav. Rev..

[19] Bjorlie K., Forbush K.T., Chapa D.A.N., Richson B.N., Johnson S.N., Fazzino T.L. (2022). Hyper-Palatable Food Consumption during Binge-Eating Episodes: A Comparison of Intake during Binge Eating and Restricting. Int. J. Eat. Disord..

[20] van Strien T., Gibson E.L., Baños R., Cebolla A., Winkens L.H.H. (2019). Is Comfort Food Actually Comforting for Emotional Eaters? A (Moderated) Mediation Analysis. Physiol. Behav..

[21] Dorton H.M., Luo S., Monterosso J.R., Page K.A. (2018). Influences of Dietary Added Sugar Consumption on Striatal Food-Cue Reactivity and Postprandial GLP-1 Response. Front. Psychiatry.

[22] Temple J.L. (2016). Behavioral Sensitization of the Reinforcing Value of Food: What Food and Drugs Have in Common. Prev. Med. (Baltim.).

[23] Demeke S., Rohde K., Chollet-Hinton L., Sutton C., Kong K.L., Fazzino T.L. (2022). Change in Hyper-Palatable Food Availability in the US Food System over 30 Years: 1988 to 2018. Public Health Nutr..

[24] Swinburn B., Kraak V., Rutter H., Vandevijvere S., Lobstein T., Sacks G., Gomes F., Marsh T., Magnusson R. (2015). Strengthening of Accountability Systems to Create Healthy Food Environments and Reduce Global Obesity. Lancet.

[25] Monteiro C.A., Moubarac J.C., Cannon G., Ng S.W., Popkin B. (2013). Ultra-Processed Products Are Becoming Dominant in the Global Food System. Obes. Rev..

[26] Qi Q., Chu A.Y., Kang J.H., Huang J., Rose L.M., Jensen M.K., Liang L., Curhan G.C., Pasquale L.R., Wiggs J.L. (2014). Fried Food Consumption, Genetic Risk, and Body Mass Index: Gene-Diet Interaction Analysis in Three US Cohort Studies. BMJ (Online).

[27] Pepino M.Y., Mennella J.A. (2012). Habituation to the Pleasure Elicited by Sweetness in Lean and Obese Women. Appetite.

[28] Fazzino T.L., Rohde K., Sullivan D.K. (2019). Hyper-Palatable Foods: Development of a Quantitative Definition and Application to the US Food System Database. Obesity.

[29] Fazzino T.L., Dorling J.L., Apolzan J.W., Martin C.K. (2021). Meal Composition during an Ad Libitum Buffet Meal and Longitudinal Predictions of Weight and Percent Body Fat Change: The Role of Hyper-Palatable, Energy Dense, and Ultra-Processed Foods. Appetite.

[30] Moraes C.E.F., Mourilhe C., Freitas S.R., Veiga G.V., Marcus M.D., Appolinário J.C. (2020). Cross-Cultural Adaptation of the Brazilian Version of the Questionnaire on Eating and Weight Patterns-5 (QEWP-5). Trends Psychiatry Psychother..

[31] De Moraes C.E.F., Mourilhe C., da Veiga G.V., de Freitas S.R., Luiz R.R., Hay P., Appolinario J.C. (2021). Concurrent Validity of the Brazilian Portuguese Version of the Questionnaire on Eating and Weight Patterns-5 (QEWP-5) in the General Population. Eat. Behav..

[32] First M.D., Spitzer R.L., Gibbon M., Williams J.B.W. (1995). The Structured Clinical Interview for DSM-IV AXIS I Disorders—Patient Edition (SCID-I/P, Version 2.0).

[33] American Psychiatric Association (2013). Diagnostic and Statistical Manual of Mental Disorders/DSM-5.

[34] Rohde P., Lewinsohn P.M., Seeley J.R. (1997). Comparability of Telephone and Face-to-Face Interviews in Assessing Axis I and II Disorders. Am. J. Psychiatry.

[35] Barufaldi L.A., Abreu G.D.A., da Veiga G.V., Sichieri R., Kuschnir M.C.C., Cunha D.B., Pereira R.A., Bloch K.V. (2016). Software to Record 24-hour Food Recall: Application in the Study of Cardiovascular Risks in Adolescents. Rev. Bras. Epidemiol..

[36] Instituto Brasileiro de Geografia e Estatística—IBGE (2011). Pesquisa de Orçamentos Familiares 2008–2009: Tabela de Composição Nutricional Dos Alimentos Consumidos No Brasil.

[37] Stunkard A. (1959). Eating Patterns and Obesity. Psychiatr. Q..

[38] Fairburn C., Cooper Z., O’Connor M., Fairburn C.G. (2008). Eating Disorder Examination—(Edition 16.0D).

[39] Raymond N.C., Peterson R.E., Bartholome L.T., Raatz S., Jensen M.D., Levine J.A. (2012). Comparisons of Energy Intake and Energy Expenditure in Obese Women with and Without Binge Eating Disorder. Obesity (Silver Spring).

[40] World Health Organization (2018). BMI Classification.

[41] Sichieri R., Bezerra I.N., Araújo M.C., de Moura Souza A., Yokoo E.M., Pereira R.A. (2015). Major Food Sources Contributing to Energy Intake—A Nationwide Survey of Brazilians Aged 10 Years and Older. Br. J. Nutr..

[42] Rodrigues R.M., Souza A.D.M., Bezerra I.N., Pereira R.A., Yokoo E.M., Sichieri R. (2021). Most Consumed Foods in Brazil: Evolution between 2008–2009 and 2017–2018. Rev. Saude Publica.

[43] Monteiro C.A., Cannon G., Moubarac J.C., Levy R.B., Louzada M.L.C., Jaime P.C. (2018). The Un Decade of Nutrition, the NOVA Food Classification and the Trouble with Ultra-Processing. Public Health Nutr..

[44] Bartholome L.T., Raymond N.C., Lee S.S., Peterson C.B., Warren C.S. (2006). Detailed Analysis of Binges in Obese Women With Binge Eating Disorder: Comparisons Using Multiple Methods of Data Collection. Int. J. Eat Disord..

[45] Mourilhe C., de Moraes C.E.F., da Veiga G.V., da Luz F.Q., Pompeu A., Nazar B.P., Coutinho E.S.F., Hay P., Appolinario J.C. (2021). An Evaluation of Binge Eating Characteristics in Individuals with Eating Disorders: A Systematic Review and Meta-Analysis. Appetite.

[46] Nogueira Bezerra I., de Carvalho Gurgel A.O., Bastos Barbosa R.G., Bezerra da Silva G. (2018). Dietary Behaviors Among Young and Older Adults in Brazil. J. Nutr. Health Aging.

[47] Lopes M.S., Caiaffa W.T., Andrade A.C.D.S., Malta D.C., Barber S., Friche A.A.D.L. (2020). Disparities in Food Consumption between Economically Segregated Urban Neighbourhoods. Public Health Nutr..

[48] Antunes A.B.S., Cunha D.B., Baltar V.T., Steluti J., Pereira R.A., Yokoo E.M., Sichieri R., Marchioni D.M. (2021). Dietary Patterns of Brazilian Adults in 2008–2009 and 2017–2018. Rev. Saude Publica.

[49] de Paula Costa D.V., Lopes M.S., de Deus Mendonça R., Malta D.C., de Freitas P.P., Lopes A.C.S. (2021). Food Consumption Differences in Brazilian Urban and Rural Areas: The National Health Survey. Ciênc. Saúde Colet..

[50] Sproesser G., Ruby M.B., Arbit N., Akotia C.S., dos Santos Alvarenga M., Bhangaokar R., Furumitsu I., Hu X., Imada S., Kaptan G. (2022). Similar or Different? Comparing Food Cultures with Regard to Traditional and Modern Eating across Ten Countries. Food Res. Int..

[51] Forney K.J., Holland L.A., Joiner T.E., Keel P.K. (2015). Determining Empirical Thresholds for “Definitely Large” Amounts of Food for Defining Binge-Eating Episodes. Eat Disord..

[52] Bezerra I.N., Goldman J., Rhodes D.G., Hoy M.K., de Moura Souza A., Chester D.N., Martin C.L., Sebastian R.S., Ahuja J.K., Sichieri R. (2014). Difference in Adult Food Group Intake by Sex and Age Groups Comparing Brazil and United States Nationwide Surveys. Nutr. J..

[53] Bartholome L.T., Peterson R.E., Raatz S.K., Raymond N.C. (2013). A Comparison of the Accuracy of Self-Reported Intake with Measured Intake of a Laboratory Overeating Episode in Overweight and Obese Women with and without Binge Eating Disorder. Eur. J. Nutr..

[54] Forbush K.T., Hunt T.K. (2014). Characterization of Eating Patterns among Individuals with Eating Disorders: What Is the State of the Plate?. Physiol. Behav..

[55] Lopes T.S., Luiz R.R., Hoffman D.J., Ferriolli E., Pfrimer K., Moura A.S., Sichieri R., Pereira R.A. (2016). Misreport of Energy Intake Assessed with Food Records and 24-h Recalls Compared with Total Energy Expenditure Estimated with DLW. Eur. J. Clin. Nutr..

[56] Burrows T.L., Ho Y.Y., Rollo M.E., Collins C.E. (2019). Validity of Dietary Assessment Methods When Compared to the Method of Doubly Labeled Water: A Systematic Review in Adults. Front. Endocrinol. (Lausanne).

[57] Castetbon K., Vernay M., Malon A., Salanave B., Deschamps V., Roudier C., Oleko A., Szego E., Hercberg S. (2009). Dietary Intake, Physical Activity and Nutritional Status in Adults: The French Nutrition and Health Survey (ENNS, 2006–2007). Br. J. Nutr..

[58] Instituto Brasileiro de Geografia e Estatística—IBGE (2011). Pesquisa de Orçamentos Familiares 2008–2009: Análise Do Consumo Pessoal No Brasil.

[59] Foster E., Lee C., Imamura F., Hollidge S.E., Westgate K.L., Venables M.C., Poliakov I., Rowland M.K., Osadchiy T., Bradley J.C. (2019). Validity and Reliability of an Online Self-Report 24-h Dietary Recall Method (Intake24): A Doubly Labelled Water Study and Repeated-Measures Analysis. J. Nutr. Sci..

[60] Verly Junior E., Marchioni D.M., Araujo M.C., de Carli E., de Oliveira D.C.R.S., Yokoo E.M., Sichieri R., Pereira R.A. (2021). Evolution of Energy and Nutrient Intake in Brazil between 2008–2009 and 2017–2018. Rev. Saude Publica.

[61] Bes-Rastrollo M., Sanchez-Villegas A., Basterra-Gortari F.J., Nunez-Cordoba J.M., Toledo E., Serrano-Martinez M. (2010). Prospective Study of Self-Reported Usual Snacking and Weight Gain in a Mediterranean Cohort: The SUN Project. Clin. Nutr..

